# Deep learning-based sleep stage classification with cardiorespiratory and body movement activities in individuals with suspected sleep disorders

**DOI:** 10.1038/s41598-023-45020-7

**Published:** 2023-10-18

**Authors:** Seiichi Morokuma, Toshinari Hayashi, Masatomo Kanegae, Yoshihiko Mizukami, Shinji Asano, Ichiro Kimura, Yuji Tateizumi, Hitoshi Ueno, Subaru Ikeda, Kyuichi Niizeki

**Affiliations:** 1 Department of Health Sciences, Graduate School of Medical Sciences, Kyushu University, Fukuoka, Japan; 2 Tokorozawa Respiratory Clinic, Tokorozawa, Japan; 3 Health Sensing Co. Ltd., Tokyo, Japan; 4 Department of Electrical Engineering, National Institute of Technology, Tokyo College, Tokyo, Japan; 5 Tokyo Information Design Professional University, Tokyo, Japan; 6 Department of Biosystems Engineering, Graduate School of Science and Engineering, Yamagata University (Emeritus), Yonezawa, Japan

**Keywords:** Health care, Sleep disorders

## Abstract

Deep learning methods have gained significant attention in sleep science. This study aimed to assess the performance of a deep learning-based sleep stage classification model constructed using fewer physiological parameters derived from cardiorespiratory and body movement data. Overnight polysomnography (PSG) data from 123 participants (age: 19–82 years) with suspected sleep disorders were analyzed. Multivariate time series data, including heart rate, respiratory rate, cardiorespiratory coupling, and body movement frequency, were input into a bidirectional long short-term memory (biLSTM) network model to train and predict five-class sleep stages. The trained model's performance was evaluated using balanced accuracy, Cohen's κ coefficient, and F1 scores on an epoch-per-epoch basis and compared with the ground truth using the leave-one-out cross-validation scheme. The model achieved an accuracy of 71.2 ± 5.8%, Cohen's κ of 0.425 ± 0.115, and an F1 score of 0.650 ± 0.083 across all sleep stages, and all metrics were negatively correlated with the apnea–hypopnea index, as well as age, but positively correlated with sleep efficiency. Moreover, the model performance varied for each sleep stage, with the highest F1 score observed for N2 and the lowest for N3. Regression and Bland–Altman analyses between sleep parameters of interest derived from deep learning and PSG showed substantial correlations (*r* = 0.33–0.60) with low bias. The findings demonstrate the efficacy of the biLSTM deep learning model in accurately classifying sleep stages and in estimating sleep parameters for sleep structure analysis using a reduced set of physiological parameters. The current model without using EEG information may expand the application of unobtrusive in-home monitoring to clinically assess the prevalence of sleep disorders outside of a sleep laboratory.

## Introduction

Sleep quality is an important determinant of human health and performance^1^. Poor sleep quality is associated with stress, anxiety, daytime sleepiness, and comorbidities, such as obesity^2^, hypertension^3^, diabetes^4^, and brain dysfunction^5^. During a sleep period, there are two types of sleep: non-rapid eye movement (N1 or N2: light sleep and N3: deep sleep) and rapid eye movement (REM) sleep. The function of the alterations between these two types of sleep is not fully understood; however, irregular sleeping patterns are known to be associated with sleep disorders. Sleep stage classification is vital for assessing sleep quality and diagnosing sleep disorders. Currently, the diagnosis of sleep disorders relies on overnight polysomnography (PSG) in a sleep laboratory. Although PSG is considered the gold standard for assessing sleep quality based on stages, it requires not only multiple physiological signals, including electroencephalography (EEG), electrooculography (EOG), chin and leg electromyography (EMG), electrocardiography (ECG), oxygen saturation (SpO_2_), and airflow, but also complex, obtrusive procedures and expensive equipment; this fact makes PSG impractical for general use in nursing homes and community health centers.

In recent years, machine learning techniques have gained significant traction because of their potential to enable the automatic classification of sleep stages from multimodal physiological data without EEG recordings^6^. For practical use, especially in low-resource settings, it is desirable to use a reduced set of input parameters as long as they contain information related to cortical activity during sleep. Cardiorespiratory signals, which are less obtrusive and relatively easy to acquire with simplified equipment, have been used for the prediction of the sleep stage^7–9^. During sleep, progression from the lighter N1 stage to the deep N3 stage is accompanied by a gradual increase in parasympathetic modulation, which leads to a lower heart rate (HR), increased vagally mediated heart rate variability (HRV), and more stable respiration. REM sleep is characterized by sympathetic dominance and suppression of the parasympathetic tone, producing sudden and abrupt changes in HR and erratic (irregular) breathing. Several studies have demonstrated the feasibility of cardiorespiratory signals and/or HRV features in predicting sleep stages ^7,10,11^. Additional insights have been provided by investigating the mutual interactions of cardiorespiratory oscillations^12–14^. Studies analyzing cardiorespiratory interactions during sleep have shown that cardiorespiratory phase coupling is strongly affected during sleep apnea events^15^ and that the temporal dynamics of slow-wave cortical activity during sleep are correlated with cardiorespiratory phase coupling^16^. These findings suggest that incorporating cardiorespiratory signals into the machine-learning model may improve the classification accuracy of the sleep stage. Accordingly, this study aimed to evaluate the performance of deep learning-based sleep stage classification with a smaller number of physiological parameters derived from cardiorespiratory signals and body movements in participants with suspected sleep disorders.

## Methods

### Participants

Untreated participants with suspected sleep disorders were recruited from a local neighborhood in Tokorozawa City, Japan. All participants underwent overnight PSG in the sleep laboratory at the Tokorozawa Respiratory Clinic between July and December 2022. The procedures and protocols were approved by the Institutional Review Board of Kyushu University (No. 21126-00) and conformed to the Declaration of Helsinki. All participants provided informed consent to participate in the study after receiving a full written and oral explanation of the procedures.

### Polysomnography

Before PSG measurement, participant demographics, including age, sex, height, weight, and body mass index (BMI), were obtained. Overnight PSG was performed using the Philips Respironics G3 sleep diagnostic system at an ambient temperature of 22–24 °C with external stimuli minimized. All the PSG-recorded signals, comprising ECG, EOG, and anterior tibial EMG; an airflow signal recorded through pressure and thermal sensors; and SpO_2_ measured with pulse oximetry were stored in EDF files through the PSG monitoring software “Sleepware G3.” EEG, EOG, EMG, and airflow signals were recorded at a sampling frequency of 200 Hz, and ECG was recorded at a sampling frequency of 500 Hz. Recorded signals in the EDF files were transferred to LabChart 8 analysis software (ADInstruments Japan, Nagoya) and then exported to text files for analysis. After the PSG recording, the metric AHI was calculated as the number of apnea and hypopnea events divided by the TST. Apnea events were identified using the nasal cannula airflow signal as periods lasting more than 10 s with flow less than 10% of the pre-event baseline. Hypopnea events were identified as a decrease in the airflow signal by ≥ 30% of the pre-event baseline with a duration of ≥ 10 s in association with the presence of ≥ 3% oxygen desaturation from the pre-event baseline^17^.

### Data processing

Four parameters—HR, respiratory rate (RR), cardiorespiratory coupling index (λ), and body movement frequency (BMF)—were used as inputs to the deep learning model for the inference of sleep stages. For this purpose, ECG, respiratory flow, and leg EMG signals were extracted from the recorded PSGs. The beat-to-beat R-R interval (RRI) of the ECG wave was calculated as the duration between successive R peaks of the ECG signal. The RRIs were then resampled at 10 Hz using the spline interpolation method to obtain RRIs with equidistant time steps. Before resampling, the calculated RRIs were inspected visually, and outliers were deleted. The HR was computed as the inverse of RRI (in beats/min). The respiratory signal was sampled at a frequency of 10 Hz and filtered by a second-order band-pass Butterworth filter with a frequency range of 0.05–0.6 Hz. The power spectral density (PSD) of the filtered respiratory signal was then obtained from a 20 s window using continuous wavelet transform (CWT). We used a complex Morlet wavelet basis function to obtain a CWT of the respiratory signal. The respiratory frequency (f_R_) was assessed by determining the most dominant peak in the PSD. The RR was f_R_ time 60 (in breaths/min).

To extract respiratory sinus arrhythmia (RSA), resampled RRIs were filtered by the band-pass filter with a frequency range of ± 50% of the dominant frequency of respiration. The cardiorespiratory coupling index λ was obtained as the phase coherence between the RSA and respiration. The details of the calculation of λ are described elsewhere^14^. Briefly, the instantaneous phases of RSA and respiration were calculated using an analytic signal approach^18^. In a complex plane, the angle between the real signal and its Hilbert transform yields an instantaneous phase. Phase coherence can be computed by measuring the invariance of the phase difference between RSA and respiration in a given time window per the following equation^17^: $\lambda (t_{k} ) = \left| \frac{1}{N} \right.\sum\limits_{j = k - N}^{k} {\left. {e^{{i\psi (t_{k} )_{j} }} } \right|}^{2}$, where N denotes the number of consecutive data samples in the computation window and *ψ* is the phase difference between RSA and respiration at a discrete time *t*_*k*_.

EMG signals from the anterior tibialis muscles were chosen as indicators of body movements, which are generally considered characteristics of arousal during sleep. After filtering by band-pass Butterworth filter with a frequency range of 1–30 Hz, EMG signals were normalized. Then, the onset timing of the body movement was detected when the normalized EMG signal exceeded a preset threshold. The threshold was set to three times the SD of the baseline EMG amplitude. If body movement was detected in a time window, the number of body movements was set to one; otherwise, it was set to zero.

All the parameters were calculated from 10 s time windows with a sliding period of 5 s and then moving averaged by applying a median filter with a 30 s window to align the time series data corresponding to the sleep stage label in the PSG records. Therefore, HR, RR, and λ represent the respective mean values of the six data points calculated every 5 s, and BMF represents the mean number of body movements in the duration of 30 s in the unit of s^−1^.

**Figure 1:**
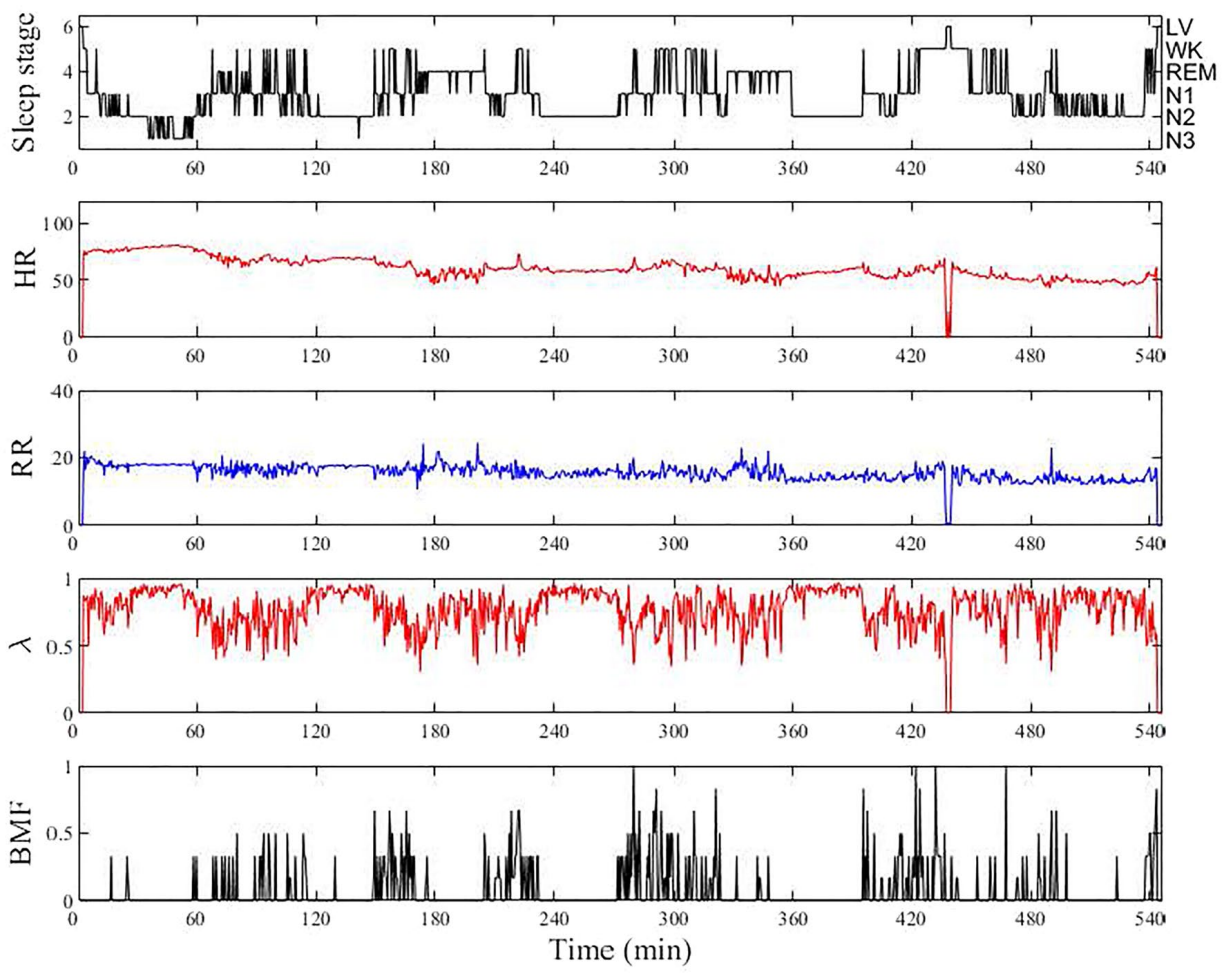
Example of input data profiles analyzed in a 47-year-old man with an AHI of 54.6, along with the sleep staging labels. From top to bottom, annotated sleep stage by a human expert, HR, RR, λ, and BMF. Each parameter was calculated for every 10 s time window with a shifting step of 5 s and then aligned at 30 s intervals to correspond to the sleep staging label. Note that the LV in the sleep staging label indicates “getting out of bed” (see Data processing).

An example of the overnight parameter profiles, along with the sleep staging label scored by a human expert for an individual participant, is shown in Fig. 1. Sleep stages were classified according to the American Academy of Sleep Medicine^19^ as follows: wake (WK), rapid-eye-movement sleep (REM), non-rapid eye movement sleep N1, N2, and N3. Although the state of “getting out of bed” is not included in the AASM annotation rule, we considered the stage to be "getting out of bed" (referred to as LV) when ECG, EMG, and airflow signals disappeared for a period of more than 2 epochs (see Fig. 1 at around 430 min). This was done because missing signals do not provide any information to the deep learning model to learn the underlying physiological relationship between input variables and sleep stages. Additionally, four epochs of the LV stage were added at the beginning and end of each analyzed dataset (added zero data) and PSG staging labels (added LV label). This was done to give the model more data points to better predict the minority outcome of “LV.” The different functions of sleep stages resulted in variations in HR, RR, λ, and BMF. As sleep progressively deepened from the N1 to N3 stages, HR and RR became stable, λ stabilized at a higher level, and BMF became less frequent (Fig. 1). In contrast, during the REM stage, HR and RR fluctuated more and λ decreased while fluctuating. BMF tended to be more frequent in WK.

### Machine learning model

**Figure 2:**
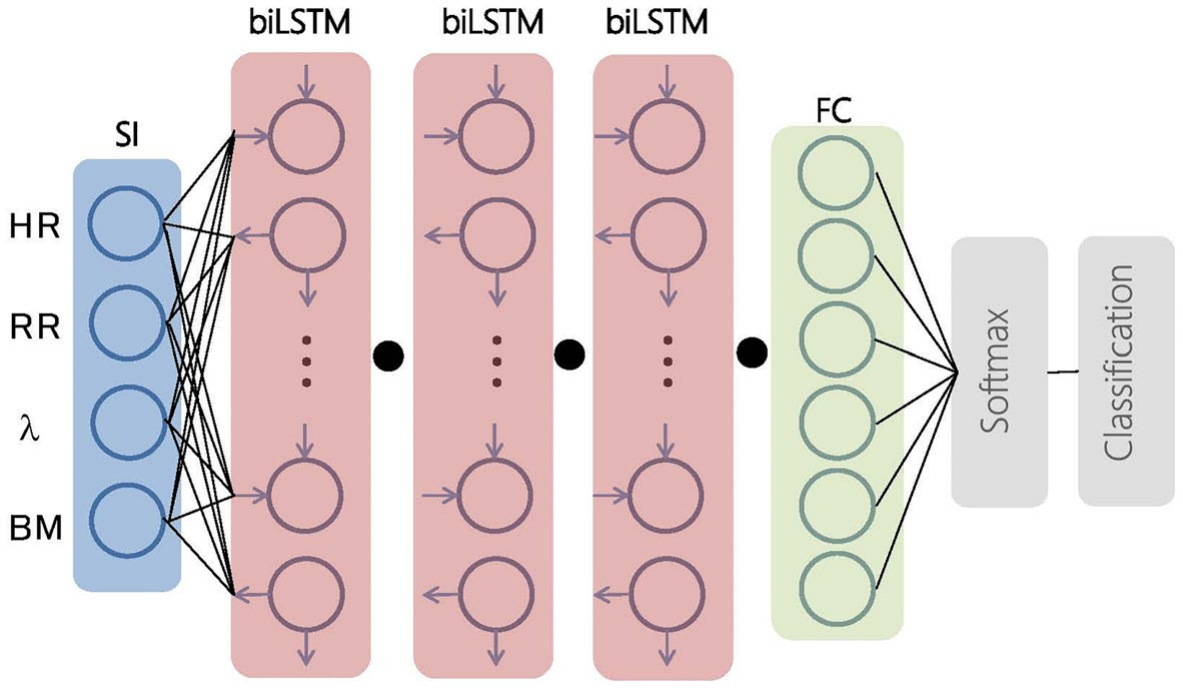
Architecture of a biLSTM deep learning neural network model for classification. Closed circles denote dropout layers. FC: fully connected layer; SI: sequence input layer.

The inference of sleep stages is done by training a deep neural network to learn the association between the multivariate time series of HR, RR, λ, and BMF and the sequence of sleep-staging labels scored by human experts. We used a biLSTM neural network to classify the sleep stages. The biLSTM architecture can learn sequence information in backward and forward directions^20^. We considered the biLSTM model suitable for predicting sleep staging because the transitions of sleep progression are known to have a temporal dependency. An overview of the model is shown in Fig. 2. The model was composed of a sequential input layer, three biLSTM layers, and a fully connected layer. Each bi-LSTM layer consisted of 128 hidden cells. A dropout layer (rate of 0.2) was added after each bi-LSTM layer to reduce overfitting. The fully connected layer consisted of six neurons corresponding to the six class probabilities of light non-REM sleep (N1, N2), deep non-REM sleep (N3), REM sleep (REM), WK, and LV. The softmax function was used as the activation function in the output of the fully connected layer, which could be used to predict the multinomial probability distribution. Finally, the classification layer classified the epochs by the epoch sleep stage based on the probability distribution.

### Training and validation

The model was trained and validated using a leave-one-out cross-validation scheme, in which each observation is considered the validation set and the remaining observations (number of observations − 1) are considered the training set. This process is repeated for the number of observations for each observation as the validation set. The training data were split into mini-batches and shorter sequences were zero-padded at the end so that all sequences had the same length. To reduce the amount of padding, sequences in the training data were sorted by sequence length before training. The model was trained with a mini-batch, the size of which was determined by the longest sequence among the entire set of input sequences. The Adam training optimizer was used to minimize the categorical cross-entropy loss function (CEL) between predicted classes and actual labels, which can be computed as: ${\text{CEL}} = - \frac{1}{N}\mathop \sum \limits_{i = 1}^{N} \mathop \sum \limits_{c = 1}^{5} P\left( {S_{i} = C_{c} } \right) \cdot {\text{ln}}\left\{ {\hat{P}\left( {S_{i} = C_{c} } \right)} \right\}$, where N is the total number of epochs, *C*_*i*_ represents the sleep stage assigned by the sequential ordinal value *i*, $P({S}_{i}={C}_{c})$ is the fraction of the sequence *S*_*i*_ scored as *C*_*c*_ by PSG measurements, and $\widehat{P}({S}_{i}={C}_{c})$ is the fraction of the sequence *S*_*i*_ predicted as* C*_*c*_ by the model. By minimizing this loss function, the model learns to assign higher probabilities to the correct class while reducing the probabilities for incorrect classes. The initial learning rate was set to 1e^−2^, which was reduced to one-hundredth of the previous rate every 50 epochs. The L2 norm-based gradient threshold method was employed to clip the gradient values that exceeded the gradient threshold of 2. The training process was terminated if there was no improvement in the test data over 50 consecutive epochs. The validation accuracy and loss function were used as stopping criteria to avoid over-fitting. The hyperparameters used in this study are summarized in Table 1. The values of the hyperparameters were selected based on several try-and-error trials. We trained the model on a machine with an NVIDIA GeForce RTX3060 GPU and 32 GB of VRAM.

**Table 1:** Ranges and selected values of hyperparameters for deep learning.

Hyperparameters	Range searched	Selected values
Number of biLSTM layer	[2,3,4]	3
Hidden units in individual biLSTM cell	[32,64,128,256]	128
Drop out rate	[0.1,0.2,0.3]	0.2
Initial learn rate	[0.001,0.01, 0.1]	0.01
Dropping of learning rate	[0.01–0.1]/50 epochs	0.01/50 epochs
L2 regularization	[0.01, 0.1, 1]	0.1
Gradient threshold	[0.1,1,2,3]	2
Mini batch size	[shortest, longest]	longest sequence
Maximum epochs	[1000,2000,3000,5000]	5000

### Data analysis

#### Evaluation of model performance

Recognizing the imbalance problem in sleep scoring data, we used the metrics of balanced accuracy, Cohen's κ, and F1 score statistic of the epoch-per-epoch agreement for sleep staging to evaluate the sleep stage classification performance of our model in comparison with the ground truth. A multiclass confusion matrix was generated to analyze the outcomes. To construct the confusion matrix, the LV class was combined with WK, and the sleep stages from N3 to N2, N1, REM, and WK were sequentially assigned ordinal values between 1 and 5. Overall performance metrics of balanced accuracy, Cohen’s κ, and F1 score were computed from the following equations^21^:

$${\text{Accuracy}} = \frac{1}{2}\left( {\frac{{\mathop \sum \nolimits_{c}^{C} TP_{c} }}{{\mathop \sum \nolimits_{c}^{C} TP_{c} + \mathop \sum \nolimits_{c}^{C} FN_{c} }} + \frac{{\mathop \sum \nolimits_{c}^{C} TN_{c} }}{{\mathop \sum \nolimits_{c}^{C} FP_{c} + \mathop \sum \nolimits_{c}^{C} TN_{c} }}} \right),$$

$${\text{Cohen's }}\kappa = \frac{{\mathop \sum \nolimits_{c}^{C} TP_{c} }}{{\mathop \sum \nolimits_{c}^{C} TP_{c} + \mathop \sum \nolimits_{c}^{C} FN_{c} }} + \frac{{\mathop \sum \nolimits_{c}^{C} TN_{c} }}{{\mathop \sum \nolimits_{c}^{C} FP_{c} + \mathop \sum \nolimits_{c}^{C} TN_{c} }} - 1,$$

$${\text{F1 score}} = \frac{{2 \cdot \mathop \sum \nolimits_{c}^{C} TP_{c} /\left( {\mathop \sum \nolimits_{c}^{C} TP_{c} + \mathop \sum \nolimits_{c}^{C} FN_{c} } \right)}}{{2 + \mathop \sum \nolimits_{c}^{C} TP_{c} /\left( {\mathop \sum \nolimits_{c}^{C} TP_{c} + \mathop \sum \nolimits_{c}^{C} FN_{c} } \right) - (\mathop \sum \nolimits_{c}^{C} TN_{c} /\left( {\mathop \sum \nolimits_{c}^{C} FP_{c} + \mathop \sum \nolimits_{c}^{C} TN_{c} } \right)}},$$

where *TP*_c_, *TN*_c_, *FP*_c_, and *FN*_c_ are true positives, true negatives, false positives, and false negatives of categorical class *c* $\in${1, 2, 3, 4, 5}, respectively, and C is the number of categorical sleep stage ( i.e., C = 5).

Performance was also analyzed on a per-class basis using the same metrics.

#### Estimation of sleep parameters

We investigated the potential of the deep-learning-based sleep stage classification in calculating sleep parameters. The following six sleep parameters were chosen: total sleep time (TST), defined as the total time of non-wake stages; sleep latency (SL), defined as the duration of time from bedtime to the onset of sleep; wake after sleep onset (WASO), calculated as time in bed (TIB)-SL-TST; percent time of REM sleep in TST (REM%); percent time of NREM sleep in TST (NREM%); and sleep efficiency (SE), defined as TST/TIB. These parameters derived from deep learning were compared with those assessed by PSG measurements.

#### Statistical analyses

The effect of AHI, age, and SE on overall balanced accuracy, Cohen's κ, and F1 score was examined using Pearson correlation coefficients. The association of AHI categories with the performance metrics was also assessed using repeated-measures one-way ANOVA with Bonferroni corrections. To compare the performance distribution of each sleep stage, the Kruskal–Wallis rank-sum test was applied to avoid the assumption of normality. Scheffé's F test was used for multiple comparisons when a statistically significant difference was observed between sleep stages. Deming regression analysis and Bland–Altman plots were used to describe the relationship and agreement between sleep parameters predicted by deep learning and those assessed by PSG measurements. The level of agreement (LoA) and mean bias for each sleep parameter were calculated. Spearman rank correlation analysis was performed between the difference and the average of sleep parameters assessed by PSG measurement and derived by deep learning to test for constant or proportional bias in the Bland–Altman plots. Data are presented as mean ± SD. All statistical tests were 2-sided, and a *P* value < 0.05 was judged to be statistically significant. Data processing and analyses were performed in MATLAB (R2022b; MathWorks) with customized scripts, a machine learning toolbox, a wavelet toolbox, and a signal processing toolbox.

## Results

The total number of participants with suspected sleep disorders was 123, including 22 females. The participants’ demographics and sleep statistics are presented in Table 2. The systolic and diastolic pressures measured just before the PSG examination were 133 ± 16 and 80 ± 11 mmHg, respectively. The mean apnea–hypopnea index (AHI) was 30.8 ± 23.2 events/h (range: 2.6–127.9); 53 participants (43.1%) had an AHI of > 30, 33 participants (26.8%) had an AHI of 15–30, 29 participants (23.6%) had an AHI of 5–15, while 8 participants (6.5%) had an AHI of < 5. There was no significant correlation between age and AHI (*r* = 0.059, *p* = 0.515, see “Supplementary file”).

**Table 2:** Demographics and sleep statistics of participants.

Parameter	Mean ± SD	Range
Age (years)	50.4 ± 13.4	19–82
BMI (kg/m^2^)	26.5 ± 5.0	15.5–45.1
SPT (min)	541 ± 32	444–614
TST (min)	448 ± 78	196–557
SL (min)	14.7 ± 23.1	0.5–181.5
SE (%)	81.1 ± 11.9	37.0–96.2
WK%	19.9 ± 11.9	4.4–63.3
REM%	11.9 ± 5.1	2.6–28.7
N1%	21.9 ± 11.3	5.8–76.8
N2%	42.4 ± 12.9	8.3–68.6
N3%	3.9 ± 4.2	0.0–16.9
WASO (min)	91.4 ± 63.3	10.8–317.5
AHI (events/h TST)	30.8 ± 23.2	2.6–127.9

Values are means ± SD. *BMI* body mass index, *SPT* sleep period time, *TST* total sleep time, *SL* sleep latency, *SE* sleep efficiency, *WK*% percent time of wake stage in TST, *REM*% percent time of rapid eye movement sleep stage in TST, *N1*% percent time of non-REM N1 sleep stage in TST, *N2*% percent time of non-REM N2 sleep stage in TST, *N3*% percent time of non-REM N3 sleep stage in TST, *WASO* wake after sleep onset, *AHI* apnea–hypopnea index.

**Figure 3:**
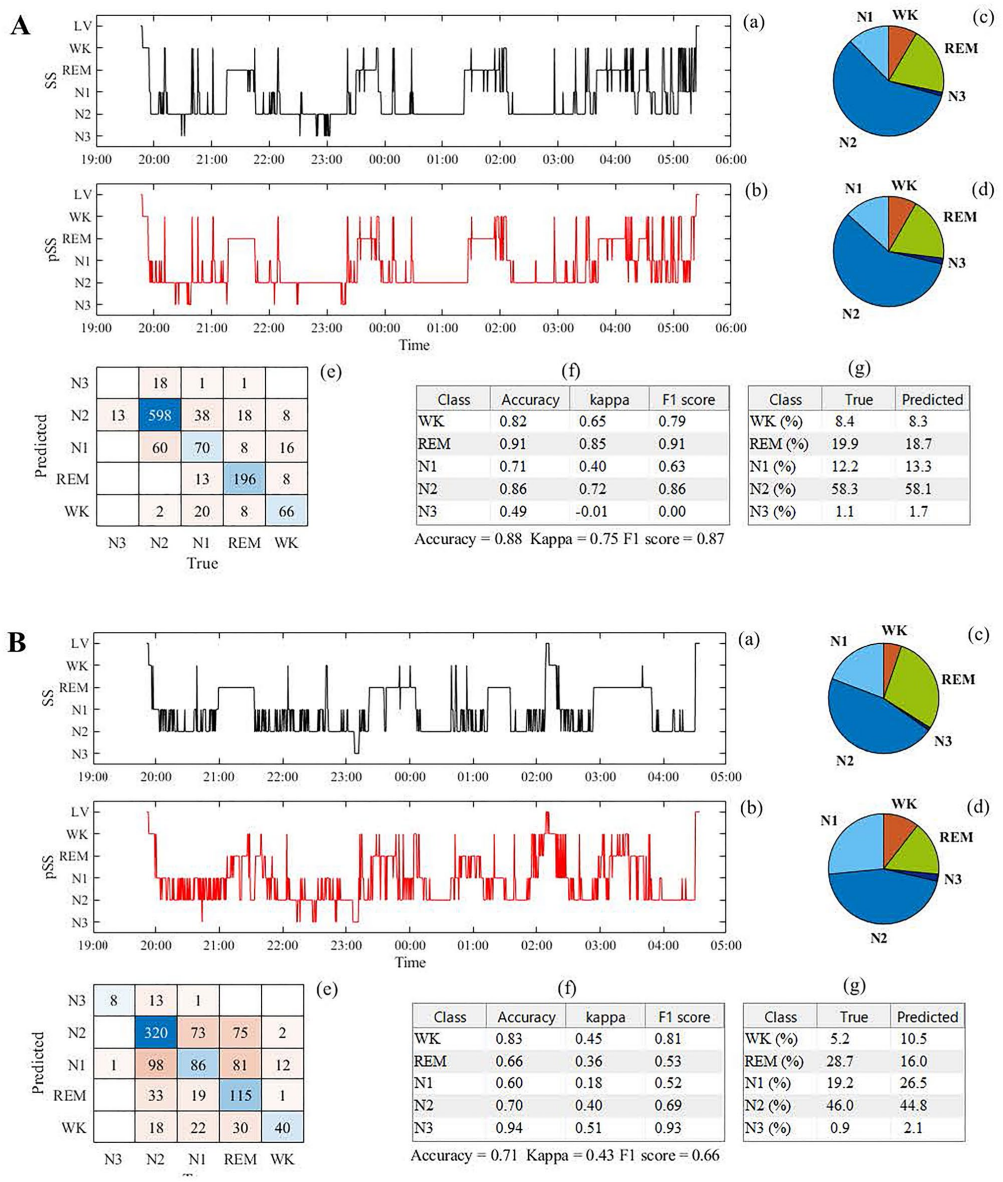
Examples of an overnight sleep stage prediction by the model in the low- (**A**) and high-AHI (**B**) participants at the same age. In both **A** and **B**, (**a**) sleep stage scored by a human expert; (**b**) predicted sleep stage by the model; (**c**) and (**d**) pie charts whose segments represent the fractions of each of the sleep stages of TIB; (**e**) confusion matrix; (**f**) balanced accuracy, Cohen’s κ, and F1 score of each of the sleep stages derived from the confusion matrix; (**g**) percentage of time spent in each sleep stage to the TIB for the ground truth (True) and prediction (Predicted). (**A**) a 37-year-old man with an AHI of 3.2, (**B**) a 37-year-old man with an AHI of 80.2. SS: sleep stage, pSS: predicted sleep stage, WK: wake stage, REM: rapid eye movement sleep stage, N1: non-REM N1 sleep stage, N2: non-REM N2 sleep stage, N3: non-REM N3 sleep stage.

Figure 3 shows examples of the sleep stage predicted by the model in low- (AHI = 3.2; Fig. 1a) and high-AHI participants (AHI = 80.2; Fig. 1b) of the same age. A confusion matrix was generated to evaluate the performance of the model. In the low-AHI case, the WK, REM, and N2 classes achieved greater classification performance in terms of balanced accuracy, Cohen’s κ, and F1 score metrics than the N1/N3 classes [Fig. 3A(f)]. However, the epoch-by-epoch N3 class could not be correctly estimated by the model, possibly because of the insufficient duration of N3. The fractions of each sleep stage of the TIB in the prediction almost agreed with those of the ground truth, as shown in the pie charts [Fig. 3A(c) and (d)] and table displayed in Fig. 3A(g). In this low-AHI case, the performance in balanced accuracy, Cohen's κ, and F1 score were 0.88, 0.75, and 0.87, respectively. While the accuracy, Cohen's κ, and F1 score in the high-AHI participant (0.71, 0.43, and 0.66, respectively) (Fig. 3B) were lower than those in the low-AHI participant (Fig. 3A), the structure of the sleep-stage transition pattern was captured well.

**Figure 4:**
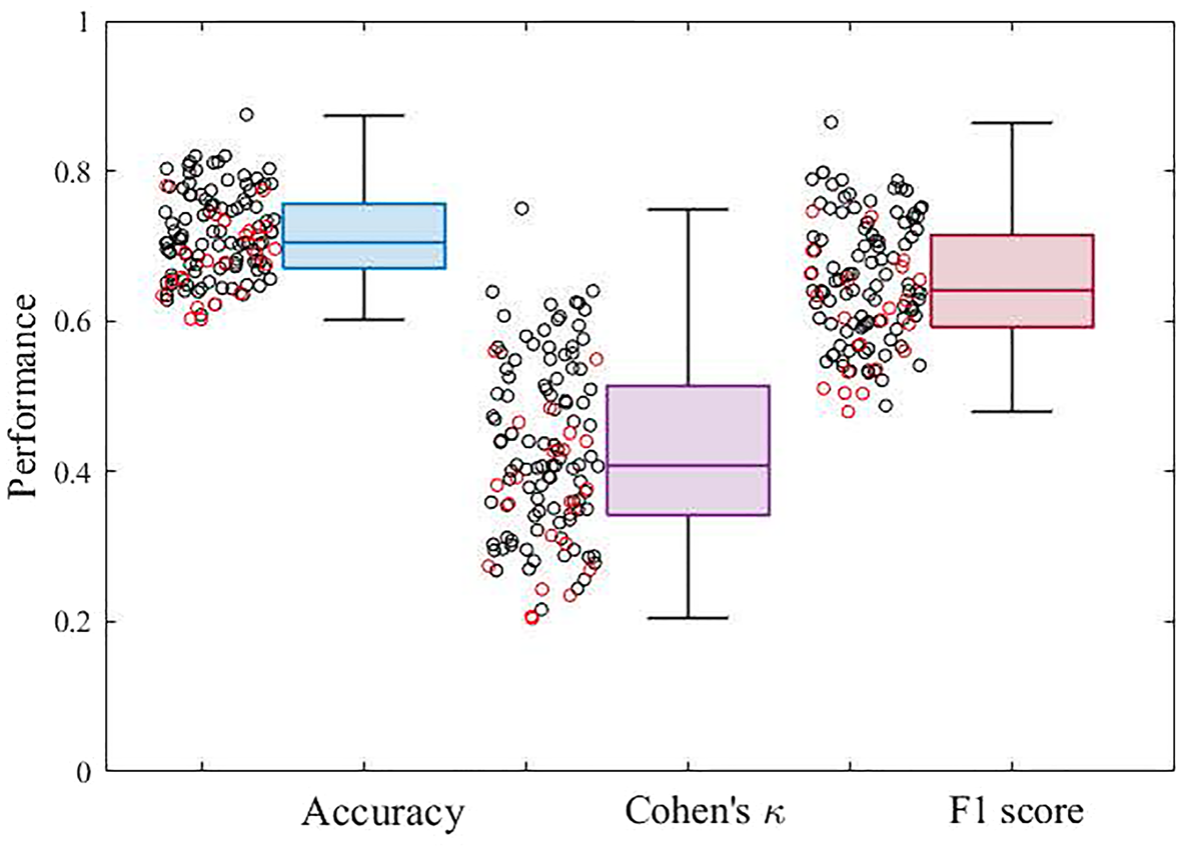
Box plot showing the distribution of overall performance in terms of balanced accuracy, Cohen's κ, and F1 score. The box plot displays the 25th percentile, median, and 75th percentile. Whiskers extend to the minimum and maximum values. Black and red open circles denote male and female participants, respectively.

**Figure 5:**
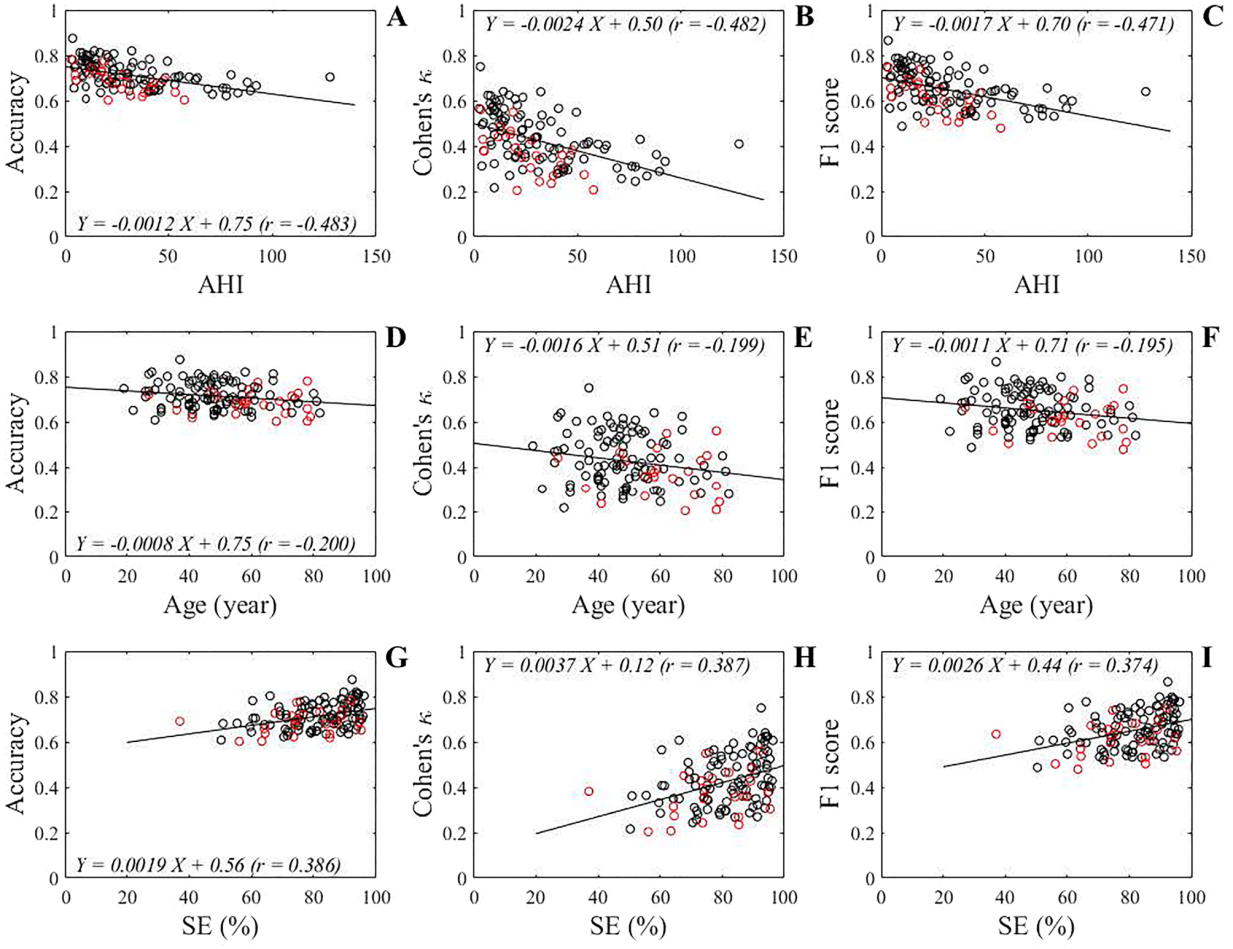
Scatter plots of overall performance in terms of balanced accuracy, Cohen's κ, and F1 score against AHI (**A**, **B**, **C**), age (**D**, **E**, **F**), and SE (**G**, **H**, **I**) of participants. Black and red open circles denote male and female participants, respectively. Regression lines are displayed with solid lines. Linear regression equations with a correlation coefficient (*r*) for each relation are indicated. AHI: apnea hypopnea index; SE: sleep efficiency.

**Figure 6:**
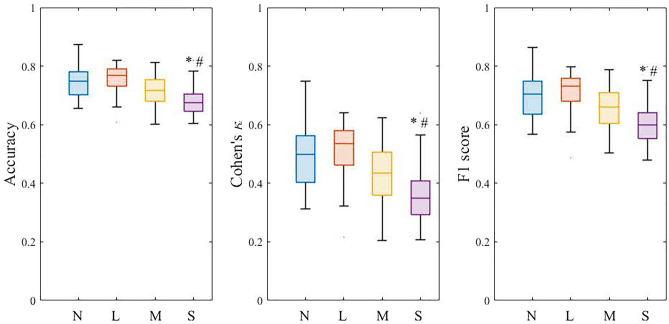
Box plot showing the distribution of overall performance in terms of balanced accuracy, Cohen's κ, and F1 score, split across AHI categories of normal (N: AHI < 5), mild (L: AHI 5–15), moderate (M: AHI  15–30), and severe (S: AHI > 30). The box plot displays the 25th percentile, median, and 75th percentile. Whiskers extend to the minimum and maximum values. ^*^P < 0.05 vs. N, ^#^*P* < 0.05 vs. L.

Figure 4 shows the box plot distribution of performance metrics reported in balanced accuracy, Cohen's κ, and F1 score for all data. The average accuracy, Cohen's κ, and F1 score were 0.712 ± 0.058, 0.425 ± 0.115, and 0.650 ± 0.083, respectively. The relationships of the metrics with AHI, age, and SE are shown in Fig. 5. As shown in Fig. 5A,B, and C, all metrics were significantly and negatively correlated with AHI (accuracy: *r* =  − 0.483, *p* < 0.0001; Cohen's κ: *r* =  − 0.482, *p* < 0.0001; F1 score:* r* =  − 0.471, *p* < 0.0001). Although we did not observe a significant correlation between age and AHI, significant but weak negative correlations of age with accuracy (*r* =  − 0.200, *p* = 0.013), Cohen's κ (*r* =  − 0.199, *p* = 0.014), and F1 score (*r* =  − 0.195, *p* = 0.015) were detected (Fig. 5D,E, and F). On the other hand, as shown in Fig. 5G,H, and I, all metrics were positively correlated with SE (accuracy: *r* = 0.386, *p* < 0.0001; Cohen's κ: *r* = 0.387, *p* < 0.0001; F1 score:* r* = 0.374, *p* < 0.0001). When the AHI was categorized using commonly used clinical cutoff points, subjects in the severe AHI group had significantly lower performance metrics compared to the normal and mild AHI groups (*p* < 0.05), as shown in Fig. 6.

To understand whether the classification performance differed among sleep stages, per-class performance metrics were also assessed (Table 3). The differences between each of these metrics, for each sleep stage, were compared (Kruskal–Wallis rank-sum test). The accuracy and Cohen’s κ in N1 were found to be low compared with other sleep stages, while N3 was lowest in the F1 score.

**Table 3:** Detailed per-class performance metrics of balanced accuracy, Cohen’s κ, and F1 score.

Class	Accuracy	Cohen’s κ	F1 score
WK	0.714 ± 0.082	0.409 ± 0.136	0.632 ± 0.143
REM	0.707 ± 0.130	0.424 ± 0.250	0.565 ± 0.270
N1	0.582 ± 0.050^a,b^	0.147 ± 0.091^a,b^	0.460 ± 0.099^a,b^
N2	0.687 ± 0.072^c^	0.360 ± 0.146^b,c^	0.670 ± 0.112^B,c^
N3	0.624 ± 0.144^a,b,d^	0.194 ± 0.202^a,d^	0.343 ± 0.324^a,b,d^

Values are means ± SD. *WK* wake stage, *REM* rapid eye movement sleep stage, *N1* non-REM N1 sleep stage, *N2* non-REM N2 sleep stage, *N3* non-REM N3 sleep stage.

^a^*P* < 0.01 vs. WK; ^b^*P* < 0.01 vs. REM; ^c^*P* < 0.01 vs. N1; ^d^*P* < 0.01 vs. N2; ^B^*P* < 0.05 vs. REM.

**Figure 7:**
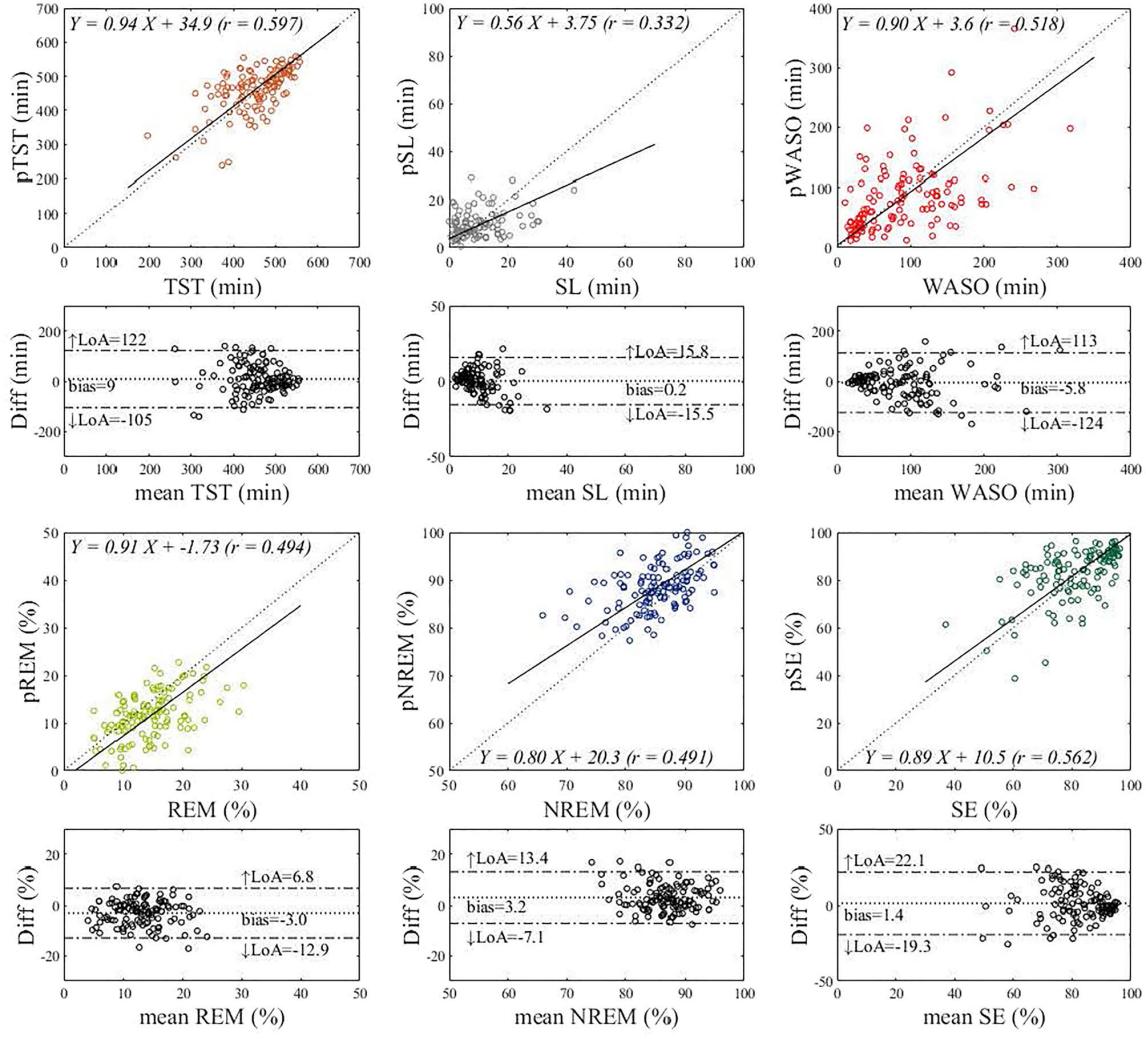
Bland–Altman analysis and Deming regression for the comparison between PSG-derived- and deep-learning-based sleep parameters. The Deming regression plot illustrates the fitted linear model (solid line) and the identity line (dotted line). Linear regression equations with correlation coefficient (*r*) for each relation are indicated. In Bland–Altman plots, 95% limits of agreement (LoA) and bias are displayed with dashed and dotted lines, respectively, together with their numerical values. The upper (↑LoA) and lower (↓LoA) LoA are presented with ± 1.96 SD. The prefix *p* of the sleep parameters means “predicted” by deep learning. TST: total sleep time; SL: sleep latency; WASO: wake after sleep onset; REM%: percent time in REM sleep in TST; NREM%: percent time in NREM sleep in TST; SE: sleep efficiency.

A pairwise comparison of major sleep parameters of TST, SL, WASO, REM%, NREM%, and SE derived from deep learning and assessed by PSG measurements is summarized in Table 4. Also, results of the Bland–Altman analysis and Deming regression for the comparison are shown in Fig. 7. Among the sleep parameters, REM% was underestimated by 3.1% and NREM% was overestimated complementary by 3.1% (*p* < 0.001), while the other four parameters were nonsignificant (TST: *p* = 0.063, SL: *p* = 0.145, WASO: *p* = 0.290, and SE: *p* = 0.095). As can be observed in Fig. 7, there were moderate and significant correlations between sleep parameters assessed by PSG measurements and those predicted by deep learning (*r* = 0.332–0.597). Spearman rank correlation analysis between the differences and means of all the Bland–Altman plots demonstrated that no proportional bias was found throughout the measurement range except the SL (TST: *p* = 0.629, SL: *p* < 0.001, WASO: *p* = 0.468, REM%: *p* = 0.563, NREM%: *p* = 0.165, SE: *p* = 0.375). The difference in SL tends to increase in the direction of underestimation when the SL is long. There was a small bias of 9 min for TST, although the LoA was wide (upper and lower LoA were 122 and − 105 min, respectively). Similar results were obtained for WASO (bias = − 5.8 min; upper and lower LoA = 113 and − 124 min, respectively). There were also small biases for REM% (− 3.0%), NREM% (3.2%), and SE (1.4%) with the LoA around − 19 to 22%.

**Table 4:** Comparisons of major sleep parameters derived from PSG and predicted by deep learning.

Parameter	PSG	Predicted	*t*	*p*
TST (min)	452 ± 67	462 ± 63	1.87	0.063
SL (min)	14.7 ± 23.1	11.3 ± 12.0	1.47	0.145
WASO (min)	91.3 ± 63.3	85.6 ± 59.8	1.06	0.290
REM%	14.6 ± 5.1	11.5 ± 4.9	6.69	< 0.001
NREM%	85.4 ± 5.0	88.5 ± 4.9	6.83	< 0.001
SE (%)	81.1 ± 11.9	82.7 ± 10.8	1.68	0.095

Values are means ± SD. *TST* total sleep time; *SL* sleep latency; *WASO* wake after sleep onset; *REM*% percent time of rapid eye movement sleep stage in TST; *NREM*% percent time of non-REM stage in TST; *SE* sleep efficiency.

## Discussion

The present study aimed to evaluate the performance of the five-class sleep stage classification by the biLSTM model with a small number of physiological parameters derived from cardiorespiratory and body movement activities. Using four physiological parameters of HR, RR, λ, and BMF, the predicted sleep stage by the biLSTM deep learning model showed substantial agreement with the ground truth, with an average accuracy of 71.2%, Cohen’s κ of 0.43, and F1 score of 0.65. The results further showed that the estimated major sleep parameters by the current model were significantly correlated with those assessed by PSG measurements.

The accuracy achieved by the proposed method was equivalent to that of previous studies by other research groups^10,22^. Radha et al.^10^ proposed an LSTM network model in which 135 features were extracted from the HRV for four-class sleep stages, and that model achieved an accuracy of 77% and Cohen's κ of 0.61. Li et al.^22^ also classified four-class sleep stages in a very large dataset using a deep convolutional neural network model combined with a support vector machine, in which cardiorespiratory coupling in the time–frequency domain and other features of sample entropy and temporal HRV measures were derived from a single ECG signal, and that model achieved an accuracy of 75.4% and Cohen's κ of 0.54. Compared with those previous models, our model showed lower performance in Cohen's κ statistic; the approach of combining N1 and N2 into one class in previous models may explain their improved performance. Thus, we evaluated the performance of N1 and N2 combined into a light sleep category. As expected, the mean Cohen’s κ statistic increased to 0.60 ± 0.10 when combined, which is comparable to the performance of previous studies^10,22^. The accuracy and F1 score also increased (accuracy = 79.7 ± 5.5%, F1 score = 0.77 ± 0.06), which was as expected since the total number of classes was reduced from five to four.

Another possible reason for the lower Cohen's κ statistic in our five-class sleep stages classification model is the small number of healthy controls enrolled. In the present study, only eight participants were categorized as having a normal AHI. Lee et al*.*^23^ proposed a hidden-Markov model, in which several waveform features were extracted from a single frontal EEG for a four-sleep stage classifier, and they observed poorer agreement in the time in deep sleep in obstructive sleep apnea (OSA) patients than in non-OSA participants. They presumed that the poor agreement in patients with OSA was due to the lower percentage of deep sleep epochs in their training set. Therefore, the performance of the present model was also examined to determine whether it depended on the AHI. Figure 5A,B, and C illustrate the negative correlation between all performance metrics and AHI, indicating decreasing performance with increasing AHI. Cohen’s κ in the severe AHI group was significantly lower than that in the normal and mild AHI groups, as shown in Fig. 6. Analogous results have also been reported by Zhang et al.^24^, who classified five sleep stages by inputting EEG data into a convolutional neural network model and observed that the achieved performance in terms of accuracy and F1 scores gradually decreased with increased AHI. Moreover, studies have documented that OSA patients have sympathetic hyperactivity^25^, which causes repeated sympathetic arousals during sleep^26^. Therefore, the lower performance in participants with higher AHI may be due to the current set of parameters used, which do not adequately describe the sleep-stage transition complexity caused by repeated arousals from sleep.

The performance metrics were positively correlated with SE (Fig. 5G,H, and I). This is to be expected as it is known that OSA patients are more likely to have poor quality of sleep^27^. We further observed significant but weak negative correlations between performance and age (Fig. 5D,E, and F). This age-related decrease in performance was probably independent of the AHI, as we did not observe any significant correlations between age and AHI. Consistently, Radha et al.^10^ also reported a decrease in performance with age, which was likely more pronounced than our results; their findings suggested that the decrease in performance in older participants might be caused by changes in autonomic function and sleep architecture with age. These findings should be confirmed in future studies.

The distribution of the performance metrics across sleep stages showed higher variability, especially for REM and N3 judging from the SD of the performance metrics (Table 3). The average accuracy and Cohen’s κ were lowest at N1, but not significantly different from those at N3. The low performance at N1 indicates the difficulty of discriminating N1 by the model. Previous studies have also pointed out that the model tended to misclassify the N1 period as N2 and WK^28–31^. When N1 and N2 were combined to form a light NREM sleep stage, accuracy and Cohen’s κ were almost the same as those at N2 in the five-class classification (accuracy = 0.68 ± 0.07, Cohen’s κ = 0.36 ± 0.14).

The average F1 score was lower at N3 than at the other sleep stages in this model, probably due to class imbalances between stages. The average duration of the N3 stage was only 3.9% of TST in the training dataset (Table 2). Generally, if labeled data are scarce, deep learning can be prone to overfitting when the learning algorithm becomes overly focused on details specific to the scarcely labeled training dataset; this factor can lead to an incorrect classification of the N3 stage in new, unseen data. Additionally, it should be noted that inter-rater agreement in scoring N3 stage has been reported to be low, with most disagreements in the scoring of stage N3 occurring due to confusion with stage N2^32^. Thus, potential bias from human experts might have affected the classification performance.

We also investigated the potential clinical efficacy of our work by comparing the sleep parameters derived from predicted sleep stages by the current model with those assessed by PSG measurements. There was a trend of proportional bias found in SL: bias increases in the direction of underestimation with longer SL. Since SL is defined as the elapsed time from the start of being on the bed to the first 30 s epoch scored as sleep, if the model makes an incorrect prediction, for instance, predicting “sleep” while the individual remains awake from the start of being on the bed, which results in a shorter SL than the time it takes to fall asleep. At present, our proposed model does not facilitate the detection of SL. Future research is warranted to investigate how SL may reliably be detected. Other sleep parameters were found to be in modest correlations with the slope of the regression line around 0.80–0.94 and the absence of proportional bias, demonstrating the capability of the current approach in estimating sleep parameters for sleep structure analysis.

In the present study, the cardiorespiratory coupling as assessed by the phase coherence (λ) between the RSA and respiration was incorporated into the model as an input. This measure has been found to preferentially reflect parasympathetic (vagal) activity^33^. The autonomic profile of sympathetic inhibition and parasympathetic activation caused an increase in λ. Therefore, it was expected that λ would vary depending on the degree of sleep depth. The correlation of EEG power and λ dynamics has been previously described, with a clear association of increasing slow wave activity with increased λ^16^. This observation suggests that there may exist a neuronal pathway that leads to cortico-cardiorespiratory coupling. However, it is unclear the extent to which λ follows a distinct pattern depending on the sleep stage. As demonstrated in the representative input data profile (Fig. 1), a stable increase in λ can be observed not only in N3 but also in the N2 sleep stage. Changes in λ as they relate to sleep stages warrant further investigation. A similar index was used by Thomas et al*.*^34^ and Li et al.^22^ to predict sleep structures from a single ECG signal; however, their coupling index was a Fourier-transform-based cross-power spectrum between HRV and ECG-derived respiration, which was calculated over an 8.5 min temporal window. The computation of the cross-spectrum assumes that the signal is stationary in the analyzed temporal window, indicating that the spectral composition does not vary over time, whereas the time domain λ can be calculated on a 30 s epoch-by-epoch basis without restrictions. Therefore, λ may have some advantages over cross-spectral analyses, especially when they are used to assess short-time-resolution data.

In summary, the present study demonstrated that the biLSTM deep learning model performed well in a five-class sleep stage classification using only four physiological parameters derived from cardiorespiratory and body movement activities in a population with suspected sleep disorders. Currently, some issues remain to be addressed, particularly regarding the limitation of model performance for participants with a high AHI score. To achieve a more accurate prediction in high-AHI participants, we need to investigate whether other cardiorespiratory parameters could provide information about sleep fragmentation associated with repeated arousal during sleep. Analyzing the performance index of sleep stage classification in terms of arousal index and periodic limb movements is also a future task. Additionally, we should consider reducing the class imbalance by recruiting more participants with a higher percentage of N3 epochs and/or healthy control participants. Considering the relatively small sample size, future studies should include a larger sample size to increase the generalization ability of the deep learning model. We expect that the biLSTM model will have better performance in automated sleep staging with more balanced training data between the classes, as this model captures the temporal dynamics of the sleep-stage transition by attending to features of the parameters in forward and backward sequences.

Predicting sleep stages from cardiorespiratory and body movement activities could simplify traditional manual scoring based on PSG in health and disease. Currently, there are some potential techniques for unobtrusively acquiring cardiorespiratory signals, such as in-bed ballistocardiographic sensors^8,35^. The results of our study will contribute to the development of daily sleep structure analyses in home environments using unobtrusive modalities, thereby aiding clinicians in assessing the prevalence of sleep disorders outside of a sleep laboratory and reducing the burden on patients to attach various sensors and electrodes.

### Supplementary Information

Supplementary Figures.

## Data Availability

The data that support the findings of this study are available from the corresponding author upon reasonable request.
