# Supplementary material for: Deep learning-based sleep stage classification with cardiorespiratory and body movement activities in individuals with suspected sleep disorders
Source: Sci Rep. 2023 Oct 18;13:17730. doi: 10.1038/s41598-023-45020-7 (PMC10584883; doi:10.1038/s41598-023-45020-7)
Supplement: Supplementary file 1 — Supplementary Figures. [file 41598_2023_45020_MOESM1_ESM.docx]

**Supplementary Figure S1**

Relationship between AHI and age in the participants.


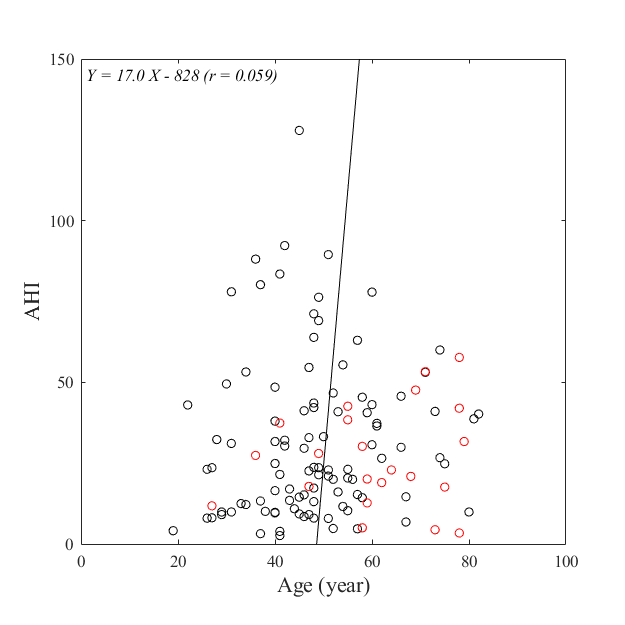


Linear regression line is displayed by solid line. Black and red open circles denote male and female participants, respectively.

**Supplementary Figure S2**

Relationship between AHI and sleep efficiency (SE) in the participants.


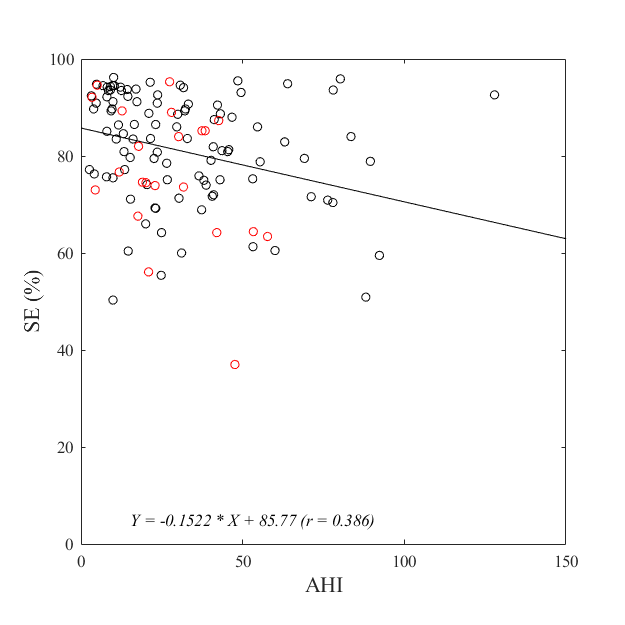


Linear regression line is displayed by solid line. Black and red open circles denote male and female participants, respectively.
